# *Saccharomyces cerevisiae* Mixed Culture of Blackberry (*Rubus ulmifolius* L.) Juice: Synergism in the Aroma Compounds Production

**DOI:** 10.1155/2014/163174

**Published:** 2014-11-23

**Authors:** Pedro Ulises Bautista-Rosales, Juan Arturo Ragazzo-Sánchez, Gabriela Ruiz-Montañez, Rosa Isela Ortiz-Basurto, Guadalupe Luna-Solano, Montserrat Calderón-Santoyo

**Affiliations:** ^1^Laboratorio Integral de Investigación en Alimentos, Instituto Tecnológico de Tepic, Avenida Tecnológico No. 2595, Colonia Lagos del Country, 63175 Tepic, NAY, Mexico; ^2^Centro de Tecnología de Alimentos, Universidad Autónoma de Nayarit, Ciudad de la Cultura “Amado Nervo”, 63155 Tepic, NAY, Mexico; ^3^Instituto Tecnologico de Orizaba, Division de Estudios de Posgrado e Investigacion, Avenida Oriente 9 No. 852, 94320 Orizaba, VER, Mexico

## Abstract

Blackberry (*Rubus* sp.) juice was fermented using four different strains of *Saccharomyces cerevisiae* (Vitilevure-CM4457, Enoferm-T306, ICV-K1, and Greroche Rhona-L3574) recognized because of their use in the wine industry. A medium alcoholic graduation spirit (<6°GL) with potential to be produced at an industrial scale was obtained. Alcoholic fermentations were performed at 28°C, 200 rpm, and noncontrolled pH. The synergistic effect on the aromatic compounds production during fermentation in mixed culture was compared with those obtained by monoculture and physic mixture of spirits produced in monoculture. The aromatic composition was determined by HS-SPME-GC. The differences in aromatic profile principally rely on the proportions in aromatic compounds and not on the number of those compounds. The multivariance analysis, principal component analysis (PCA), and factorial discriminant analysis (DFA) permit to demonstrate the synergism between the strains.

## 1. Introduction

Blackberry (*Rubus ulmifolius*) belongs to the group of fruits called berries that are considered soft fruits and include botanically different species like blueberry (*Vaccinium corymbosum, V. angustifolium, V. ashei, *and* V. macrocarpon*), strawberry (*Fragaria* sp.), gooseberry (*Ribes* spp.), and raspberry (*Rubus idaeus*) [1, 2].

The blackberry fruit has a short postharvest life of only 2-3 days and so that these fruits are used as dessert or processed foods, either canned, dried, frozen, or made into jelly, jam, or preserves. Juices are used in the manufacture of beverages (juices or concentrated pulp) and snow; however, crops are remote from the processing centers and there are few industrialization alternatives. As consequently, postharvest losses are high, making essential to develop new technologies or improve existing ones to offer alternatives of industrialization to blackberry producers. A viable alternative of industrialization is developing of median graduation spirit [1].

For alcoholic fermentation occurs favorably, it is necessary to focus on three conditions: biologics (microorganisms), physical (temperature and agitation), and chemicals (pH, nutrients) [3–7]. Furthermore, a spirit or wine of good quality must have certain sensory characteristics such as taste and smell. The flavor attributes are affected by various factors such as cultivar, the season of the crop, the variety of the fruit, temperature of fermentation, and microorganisms used, among others [3, 5, 6, 8–14].

Several factors influence the flavor profile of spirit or wine and it cannot control fully as the variability of the fruit from year to year. In counterpart, there are some factors that can be controlled, such as the type of microorganisms used in fermentation process. Each strain produces different amounts of aromatic compounds, besides that the fermentations can be carried out in pure or mixed culture. The mixed culture fermentations may have a synergistic effect, increasing the concentration of aromatic compounds produced in fermentations conducted in monoculture [9, 15–18].

The aim of this work was to evaluate the synergistic effect among* Saccharomyces cerevisiae* strains (ICV K1, Enoferm T306, Vitilevure CM4457, and Greroche Rhona L3574) in the production of aromatic compounds during fermentation of blackberry juice (*Rubus ulmifolius*).

## 2. Materials and Methods

### 2.1. Biological Material

Ripening blackberry fruits (*Rubus* sp.) from the municipality of Xalisco, Nayarit, Mexico, was used.

Also, it was used four commercial strains of* Saccharomyces cerevisiae*: ICV K1, Enoferm T306, Vitilevure CM4457, and Greroche Rhôna L3574. These yeasts are used specially in the winery and they are marketed in lyophilized form by the commercial Lallemand (Toulouse, France). These yeasts have the characteristics of being producers of aromas like floral, spices (ginger), or exotic fruits (pineapple) mainly, produce low amount foam, have short latency, killer phenotype, have rapid fermentation rate, and are resistant to ethanol concentrations of 13 to 18% v/v. These features vary according to the strain used [19].

### 2.2. Obtaining Wort

The wort used as culture medium was obtained from the extraction of juice from blackberry fruits variety Tupi, using a commercial juice extractor. Subsequently, juice was subjected to an enzymatic clarification with Cytolase PCL 5 (0.012 mL/L) (ENMEX, Tlalnepantla, State of Mexico, Mexico), which acts as degrading pectins and cellulose material. Finally, the juice was pasteurized at 65°C for 5 min. The initial concentration of sugars in the wort was 100 g of total sugars expressed as glucose per liter.

### 2.3. Fermentation

Alcoholic fermentations were performed in monoculture with four strains of* Saccharomyces cerevisiae* (ICV K1, Enoferm T306, Vitilevure CM4457, and Greroche Rhôna L3574) and then raised a factorial design (4-1), resulting in six combinations which were made binary physical mixtures of different liqueurs, considering each strain as a factor. Additionally, mixed culture fermentations were performed with the same combinations that were made in the above mixtures monoculture spirits, in order to evaluate the synergistic effect of different strains during fermentation. For spirits resulting from the fermentations in monoculture, binary physical mixtures and mixed culture, the aromatic profile was determinate.

Five fermenters were used simultaneously with a working volume of 1 liter, which include sampling, entry, and exit of gases (air and CO_2_) (with filters ACROVENT 0.2 microns), controls of agitation, and temperature. The culture medium was inoculated with 50 mg/mL yeast for monoculture, whereas for mixed culture, inoculation was conducted as shown in Table 1.

After inoculation, aeration was performed for 5 min for the incorporation of oxygen to the medium, in order to stimulate the production of biomass initially, and then the fermentation was continued in anaerobiosis, in a nitrogen atmosphere at a flow rate of 2 mL/min. The fermentation conditions were 28°C; pH was not controlled; stirring was constant (200 rpm). All fermentations were performed in triplicate. During the fermentation samples were taken, 3 during the lag phase, and once the exponential phase begin, samples were taken every 4 h, in order to monitor the concentration of biomass in the fermentation process [20].

### 2.4. Aromatic Compounds Analysis (GC-FID and GC-MS)

Aromatic composition of the alcoholic fermentations was determined using the HS-SPME-GC (Head Space-Solid Phase Micro Extraction-Gas Chromatography) technique. It was used a PDMS-DVB (polydimethylsiloxane/divinylbenzene) of 65 *μ*m SUPELCO (Bellefonte, USA).

In a vial of 10 mL capacity 5 mL of sample with 1.5 g of NaCl to induce salting out was added. It was stirred at 1000 rpm at 40°C for 20 min to establish the first thermodynamic equilibrium between the liquid phase (sample) and the gaseous phase (headspace vial). Subsequently, a second thermodynamic equilibrium was established between the gaseous phase (headspace) and the solid phase (PDMS-DVB fiber) exposing the fiber in the headspace of the vial at 40°C for 40 min. Then, the fiber was removed from the vial and the volatiles contained therein were thermally desorbed from the fiber into the injection port (split/splitness) gas chromatograph (Varian 3800 GC) equipped with a flame ionization detector (FID). Nitrogen was used as carrier gas at a flow of 2 mL/min, and hydrogen and air as combustion gasses, in a column CP-Sil 5 CB (0.25 mm × 0.25 *μ*m, with a length of 15 m) mark Varian (Palo Alto, California, USA). The injector temperature was at 250°C and the detector at 270°C. The temperature program in the column was 40°C during the first 5 min and then heated to 200°C at a rate of 2°C/min; the temperature was elevated finally to 220°C at a rate of 4°C/min.

Aromatic compounds identification was performed using a mass spectrometer (Agilent) coupled to a gas chromatograph, using the same column (CP-Sil 5 CB) temperature conditions, and flows into detection; helium gas was used as carrier, with the source temperature 230°C, 250°C cuadropole, an emission of 34.6 eV (electron Volts), scanning speed of 3 seconds, and a mass range of 35 to 350 m/s. The identification was made by a comparison with the NIST 08 database, as well as some external standards (alcohols, esters, ethers, organic acids, aldehydes, and ketones) HPLC grade (Aldrich Chemical Co., St. Louis, MO, Bedoukian Research Inc., Danbury, CT) for verification. For quantification 2-nonanol (50 mg) as internal standard was used.

### 2.5. Statistical Analysis

Two survey methods were used to data analysis, principal component analysis (PCA), and factorial discriminant analysis (DFA). A program in LabView language (Version 7.1) (National Instrument) was used for PCA and DFA [21]. The selection of aromatic compounds that allow discrimination was performed by an iterative method called “a leave-one-out” [22, 23]. This method consists in selecting each aromatic compound, observing based on a selection criterion, and testing all possible combinations of flavor compounds. The selection criteria used in this study were the change between groups. For each selected combination, the frequency of occurrence of each aromatic compound was measured and the most frequent combination of compound was proposed and used.

## 3. Results and Discussion

### 3.1. Volatile Aromatic Compounds from Blackberry Spirits

Thirty-five aromatic compounds were identified in the blackberry juice fermentations with different strains, which are listed in Table 2. In alcoholic beverages obtained from fruit juices, there are some characteristic organic compounds, which are the same as those found in non-fermented fruit juices, being rather the fractions either molar or massic of each ones the reason that exist distinct differences among these products. In various investigations in grape wine compounds have been identified: ethanol, 3-methyl-1-butanol, 3-methylbutyl acetate, hexylacetate, 3,7-dimethyl-1,6-octadien-3-ol, (E)-4-[(5R)-5,6,6-trimethylcyclohexene-1-yl] but-3-en-2-one, ethyl octanoate, 2-phenylethyl acetate, octanoic acid, and benzaldehyde, which are in various proportions depending on the cultivar, year, and season of harvest, variety of fruit, and microorganisms used [3, 6, 8, 10, 11, 24–29]. The compounds found in the different blackberry spirits produced in this essay were the same; however, they have different proportions (Table 3) depending on the strain used, which is consistent with the foregoing.

### 3.2. Differentiation of Treatments in Monoculture, Mixed Culture, and Binary Physics Mixed through PCA and DFA

Blackberry spirits were grouped in three different blocks: blackberry spirits performed in monoculture, binary physical mixture blackberry spirits performed in monoculture, and spirits performed in mixed culture.

According to PCA, 13 aromatic compounds were marked different among analyzed treatments; this was due to proportions of these ones into blackberry spirits. The compounds were ethanol, 3-methylbutyl acetate, 2-methylpropyl butanoate, 1-methylbutyl propanoate, 3, 7-dimethyl-2,6-octadien-1-ol, 3-methylbutyl butanoate, 3-methylbutyl-3-methylbutanoate, phenylmethyl propanoate, decanoic acid, 3-methylbutyl-hexanoate, butyl-2-butiryloxypropanoate, dodecanoic acid, and 5-pentyloxolan-2-one.

**Figure 1:**
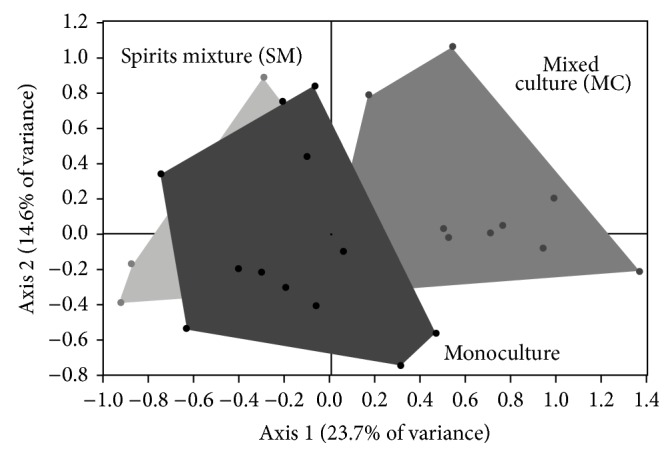
Principal component analysis map of spirit groups: monoculture, physical mixed spirits (Spirits mixture), and mixed culture.

PCA showed a total variance of 38.3% in the axis 1 and 14.6% in the axis 2 (Figure 1), where monoculture and mixed blackberry spirits blocks were overlapped almost completely; this is an expected result because the physical mixture blackberry spirits were made from product of the fermentations developed in monoculture, which explain their aromatic similarity, however, the block of fermentations in mixed culture, include all combinations, presents a confused slightly with blocks of spirits produced in monoculture processes, as the main trend was to differentiate from the other 2 groups analyzed. Coupled with this, the group of spirits obtained in mixed culture is located in the positive zone axis 1. Previous studies [21, 23, 30] have reported that the location in the axis 1 has a direct relationship with the aromatic richness of the principal compounds, which allows us to assume that there are generally higher concentration of flavor in the mixed spirit produced by mixed culture and a possible synergistic effect of the yeast on the production of aromas. This agrees with that expressed in the Table 3, which shows the average of the total concentration of principal aromatic compound in different groups of spirits.

**Figure 2:**
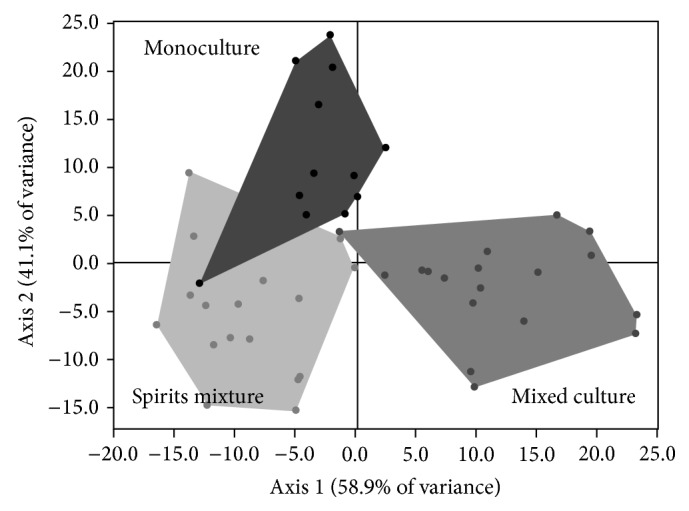
DFA map of spirit groups: monoculture, physical mixed spirits (Spirit mixture), and mixed culture.

DFA supports the differentiation of samples obtained by mixed culture from the ones obtained by monoculture (Figure 2); however, to validate this statistical analysis a confused matrix by a cross validation test should be performed, which allows to obtain the prediction error and that in this case was 16.7% according to confusion matrix in Table 4, where 40 from 48 samples were correctly classified (in bold) and 8 were classified incorrectly. It is important to note that the error was mainly due to the confusion between the products of monoculture and physical mixtures, which was expected, since they are products of the same fermentation processes; the data confusion according to Table 4 was as follows: 2 monoculture fermentations were confused with the group of mixed spirit, one mixed culture fermentation confused with another group of monoculture and another with the mixed spirit, and, finally, 3 blackberry spirit blends were confused with those in monoculture and another group with mixed culture, which can be seen more clearly in Figure 1 which is shown in the graph of the PCA.

### 3.3. Synergism between the Yeasts CM4457 and L3574

The PCA performed showed that 4 aromatic compounds had a strong influence on differentiation of treatments, which were 3-methylbutyl acetate, 1-methylbutyl propanoate, decanoic acid, and dodecanoic acid.

**Figure 3:**
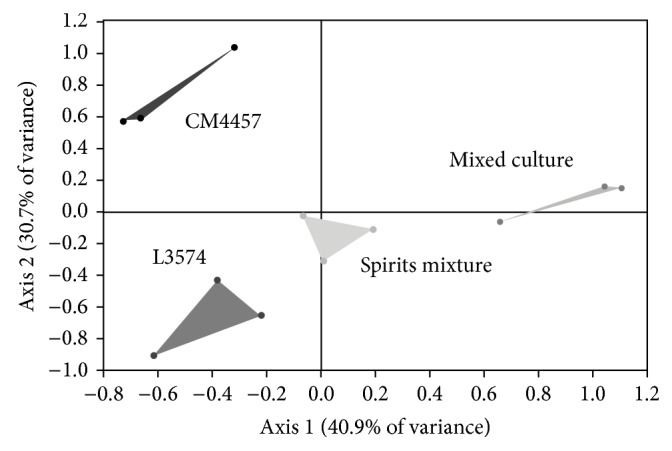
Principal component analysis map of treatments in monoculture (CM4457 and L3574), physical mixed spirits (Spirits mixture), and mixed culture with strains CM4457 and L3574.

Groups of spirits obtained in monoculture differ with respect to axis 2; however, they are located very similarly to the axis 1, mainly owing to differences not principally to aromatic richness (Figure 3). The group that represents the physical mixture of spirits is located at an intermediate point with respect to the monoculture groups, which is logical because it is representing graphically the mean between groups in monoculture. Finally, the location of culture mixed group with respect to axis 1 suggests synergy of these yeasts, causing higher production of aromas during the fermentation process. The variance indicates the total of information represented by these two axes (axis 1, 40.9%, and axis 2, 30.7%), indicating that for this analysis more than 70% of the data was considered.

**Figure 4:**
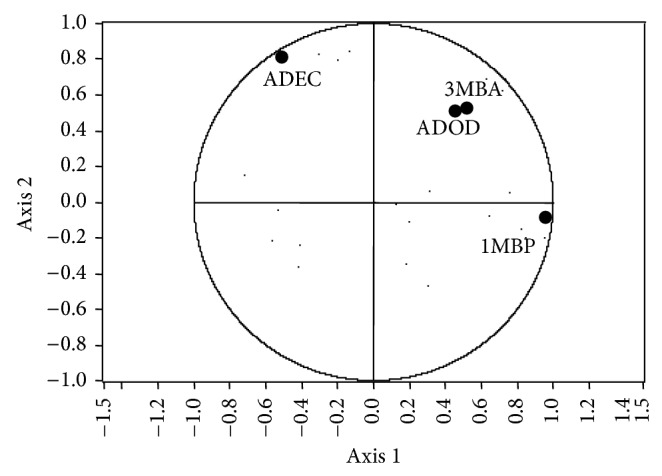
Principal component analysis score plot for treatments in monoculture, physical mixed spirits, and mixed culture with strains CM4457 and L3574 (3-methylbutyl acetate (3MBA), 1-methylbutyl propanoate (1MBP), decanoic acid (ADEC), and dodecanoic acid (ADOD)).

Ma et al. [31] mention that data in PCA are associated with *n* dimensions in the space, where *n* is a number of variables and they are reduced to some major components, which are descriptive dimensions that show the maximum variation data. According to this claim and the graph of the variables (Figure 4), the compound mainly responsible for differentiation (axis 1) is 1-methylbutyl propanoate. In regard to the axis 2, the compound is more akin to decanoic acid. If we observe the behavior of spirits in mixed culture and the mixture of spirits in monoculture, it can be seen that in the axis 1 the groups differentiate clearly, whereas in the axis 2 they are in the same direction; that is, the compound shows that the synergy is related more to the axis 1 (1-methylbutyl propanoate), since in the graph of scatter plot of PCA (Figure 3), according to the axis 1, it can be found that the mixed culture is clearly distinguishable and it is more to the right in the graph; that is, the mixed culture has a higher concentration of 1-methylbutyl propanoate as observed in Table 5, which shows the average concentration of this compound in different types of blackberry spirits.

Salmón [32] claims that when two yeasts grow and ferment in the same medium simultaneously, exchanges of metabolites among them can take place, causing sensitive modifications to kinetic properties of each of the strains, and also can cause significant changes to the end products of fermentations; that is, interactions can increase the concentration of aromatic compounds and/or make new compounds form, which is consistent with the finding in this study, as it has increased production of 1-methylbutyl propanoate and decanoic acid as shown in Table 5.

The DFA confirms the determination from PCA, since all treatments are well discriminated, besides having a prediction error of 0% (results not shown).

### 3.4. Synergism between the Yeasts ICV-K1 and T306

**Figure 5:**
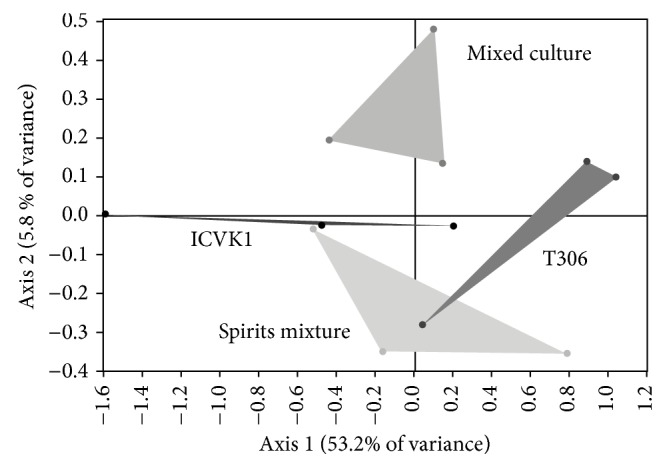
Principal component analysis map of treatments in monoculture (CM4457 and T306), physical mixed spirits (Spirits mixture), and mixed culture with strains CM4457 and T306.

**Figure 6:**
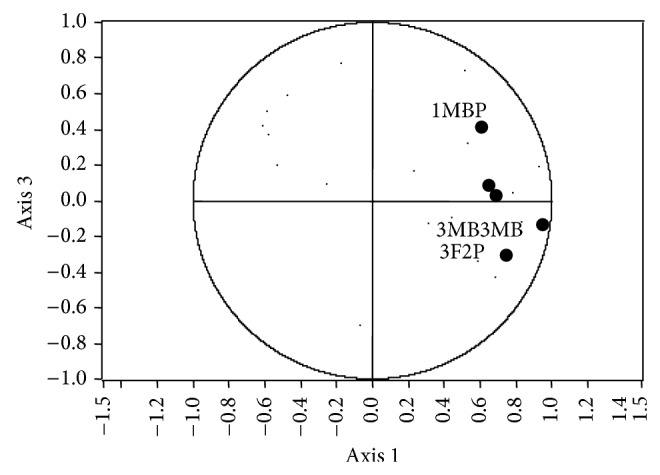
Principal component analysis score plot for treatments in monoculture, physical mixed spirits, and mixed culture with strains CM4457 and T306 (1-methylbutyl propanoate (1MBP), 3-methylbutyl-3-methylbutanoate (3MB3MB), 3-phenyl-2-propenal (3F2P), (E)-4-[(5R)-5,6,6-trimethylcyclohexene-1-il]but-3-en-2-one (BI), and ethyl dodecanoate (EL)).

According to the PCA variable selection, the main compounds that differ in the treatments in mixed culture, where the ICV K1 and T306 strains are involved, are 1-methylbutyl propanoate, 3-methylbutyl-3-methylbutanoate, 3-phenyl-2-propenal, (E)-4-[(5R)-5,6,6-trimethylcyclohexene-1-yl]but-3-en-2-one, and ethyl laurate. The PCA graph represents 59% of the variance, and it can be seen that the fermentations in mixed culture are distinct from the fermentations in monoculture and physical mixtures of spirits (Figure 5). According to the graph variables, Figure 6, in general, shows that all variables are related to the axis 1; that is, they are clustered near the right end of the axis 1, so that we can say that this axis shows the rich aroma of the principal compounds [21, 23, 30, 31, 33], which is confirmed with Table 6, where the concentrations are represented as average of the principal aromatic compounds.

Table 6 shows that treatment with higher aromatic concentration was prepared with T306 strain, which is consistent with the PCA (Figure 5), because it is the group located more to the right of the graph. According to Ma et al. [31] and to Figure 6, we can say that axis 3 has more relation with the compound 1-methylbutyl propanoate, with a variance of 5.8%. This compound shows synergism between strains and it is produced in large quantities when the fermentation is conducted in mixed culture. The DFA obtained with these principal compounds throws a prediction error of 0%; that is, no confusion among treatments and each of individuals are well classified in the group to which they belong.

### 3.5. Synergism between the Yeasts ICV-K1 and CM4457

**Figure 7:**
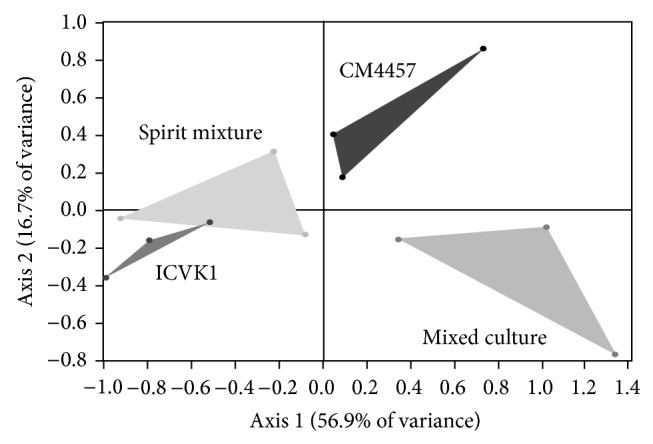
Principal component analysis map of treatments in monoculture (ICV K1 and CM4457), physical mixed spirits (Spirit mixtures), and mixed culture with strains ICV K1 and CM4457.

In this case, according to PCA, seven are the compounds that differ in the treatments: 3-methyl-1-butanol, 3-methylbutyl propanoate, 3,7-dimethyl-2,6-octadien-1-ol, 3-methylbutyl butanoate, 3-methylbutyl-3-methylbutanoate, 3-phenyl-2-propenal, and dodecanoic acid. The PCA shows a total variance from the graph of 73.6%, where in the same manner as in previous cases the axis 1 provides the rich aromatic compound of the principal compounds (Figure 7).

**Figure 8:**
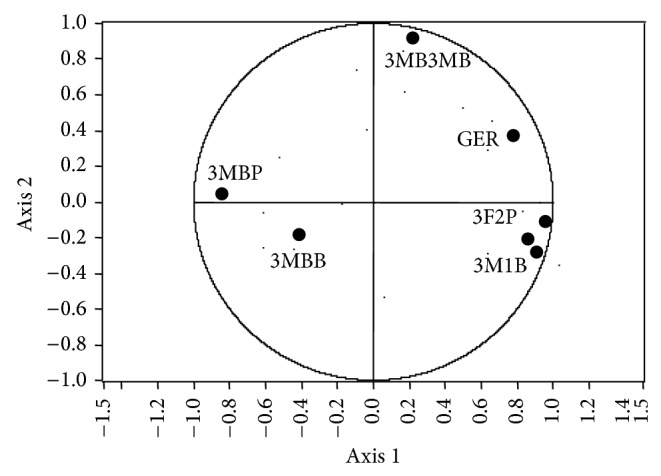
Principal component analysis score plot for treatments in monoculture, physical mixed spirits, and mixed culture with strains ICV K1 and CM4457 (3-methyl-1-butanol (3M1B), 3-methylbutyl propanoate (3MBP), 3,7-dimethyl-2,6-octadien-1-ol (GER), 3-methylbutyl butanoate (3MBB), 3-methylbutyl-3-methylbutanoate (3MB3MB), 3-phenyl-2-propenal (3F2P), and dodecanoic acid (ADOD)).

The variables graph shows that the most of the compounds are linked to the axis 1 (Figure 8), only with the exception of 3-methylbutyl-3-methylbutanoate which is mainly linked to the axis 2; comparing the location of variables in this figure, there is a behavior as follows: the variables that are to the right of the graph with respect to the origin in the axis 1 of Figure 7 would be ordered according to their concentration from left (lower concentration) to the right (higher concentration), while the variables that are left in Figure 8 with respect to origin have a behavior on reverse, since the concentration of the compounds are ordered from right (lower) to left (higher), it can be corroborated by observing Table 7, which shows the concentration of the aromatic compounds. With the foregoing and those reported by Ma et al. [31] we can say that aromatic compounds which exhibit synergism are 3-methyl-1-butanol, 3,7-dimethyl-2,6-octadien-1-ol,3-phenyl propenal, and dodecanoic acid. The DFA and cross validation exhibit confusion matrix with a prediction error of 0% (Table 8).

### 3.6. Synergism between the Yeasts ICV-K1 and L3574

**Figure 9:**
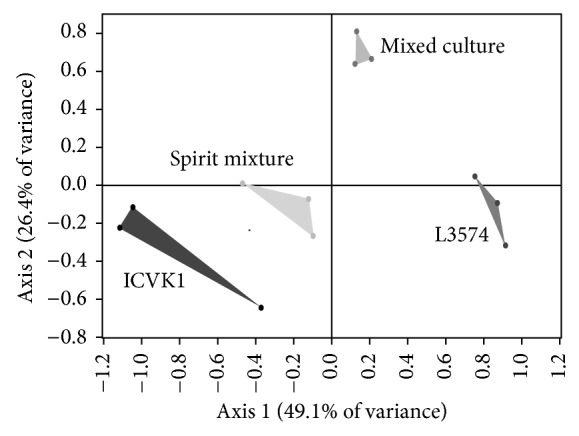
Principal component analysis map of treatments in monoculture (ICV K1 and L3574), physical mixed spirits (Spirits mixture), and mixed culture with strains ICV K1 and L3574.

The principal compounds in this analysis are 3-methyl-1-butanol, 3-methylbutyl acetate, 3-methylbutyl butanoate, dodecanoic acid, and phenylmethyl butanoate. PCA (Figure 9) shows well defined different treatments; that is, there is no confusion among them. According to the graph of variables (not shown) the compounds 3-methyl-1-butanol, 3-methylbutyl butanoate, and phenylmethyl butanoate show higher affinity for the axis 1, while 3-methylbutyl acetate and dodecanoic acid are more affined to axis 2; if it is compared with Figure 9 and the data (not shown), we can say that the latter two compounds are produced in higher quantities when the fermentation was performed in mixed culture; that is, this indicates the existence of synergism between strains. This is validated by the DFA, which gives a prediction error of 0%; that is, it does not have any problem in the differentiation of treatments.

It is interesting to note that compounds decanoic acid and dodecanoic acid inhibit the growth of some bacteria such as* Leuconostoc oenos*; in this case there is synergism among yeast in the production of dodecanoic acid which antagonizes the growth of bacteria [34], which gives stability to the final product, since, although these compounds decrease the growth of bacteria, only delays the malolactic fermentation [35] achieving a decrease the acidity and increasing volatile compounds, principally acids, esters, and alcohols [36, 37].

### 3.7. Synergism between the Yeasts L3574 and T306

**Figure 10:**
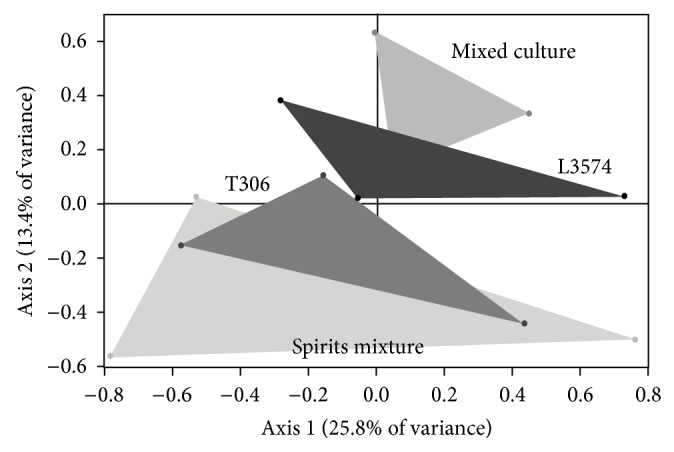
Principal component analysis map of treatments in monoculture (L3574 and T306), physical mixed spirits (Spirits mixture), and mixed culture with strains L3574 and T306. Axes 1 and 5.

PCA shows that 6 are the principal compounds which establish that the treatments are different and these are 3-methylbutyl acetate, 1-methylbutil propanoate, decanoic acid, butyl-2-butiryloxypropanoate, and 5-pentyloxolan-2-one. Comparing axes 1 and 2 of PCA, apparently no synergism exists between treatments; however, if we show the axes 1 and 5 (decanoic acid and dodecanoic acid), the mixed culture has a higher production than the other treatments (Figure 10). Compounds that are related to the axis 5 (decanoic acid and dodecanoic acid) exhibit synergism between strains L3574 and T306.

Treatments are well differentiated according to DFA and a cross validation shows a confusion matrix with zero prediction error; that is, there is no confusion between treatments.

### 3.8. Synergism between the Yeasts T306 and CM4457

According to PCA, the principal compounds are 5: benzaldehyde, 2-methylpropyl butanoate, 3,7-dimethyl-2,6-octadien-1-ol, 3-phenyl-2-propenal, butyl-2-butiryloxypropanoate, and 5-pentyloxolan-2-one.

**Figure 11:**
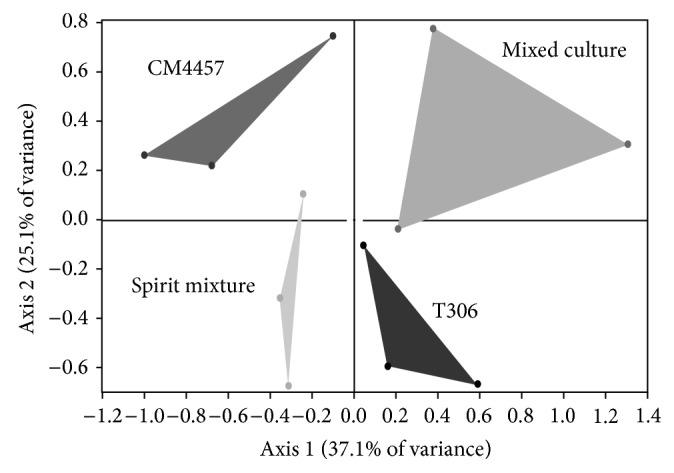
Principal component analysis map of treatments in monoculture (T306 and CM4457), physical mixed spirits (Spirit mixture), and mixed culture with strains T306 and CM4457.

**Figure 12:**
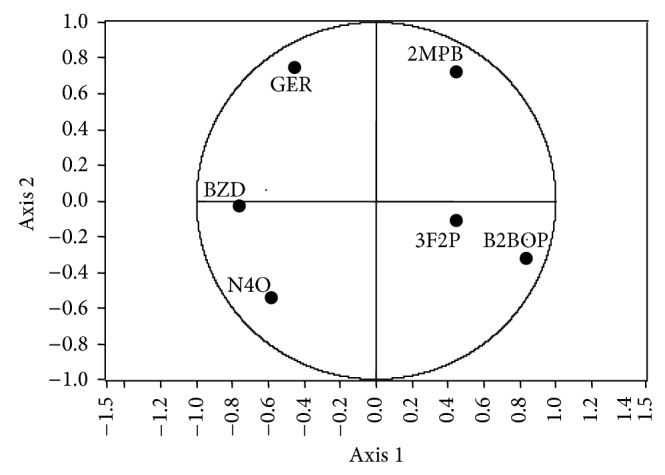
Principal component analysis score plot for treatments in monoculture, physical mixed spirits, and mixed culture with strains T306 and CM4457 (benzaldehyde (BZD), 2-methylpropyl butanoate (2MPB), 3,7-dimethyl-2,6-octadien-1-ol (GER), 3-phenyl-2-propenal (3F2P), butyl-2-buthyriloxypropanoate (B2BOP), and 5-pentiloxan-2-one (N4O)).

Figure 11 shows the graph of PCA (62.2% of variance), where the mixed culture is located to the right with respect to other treatments; that is, there is synergism according to axis 1. In accordance to variables graph (Figure 12), the compounds that have higher relation to axis 1 are benzaldehyde, 3-phenyl-2-propenal, butyl-2-butiryloxypropanoate, and 5-pentyloxolan-2-one.

In Figure 12, benzaldehyde is located on the left side of the graph (downside), which reverses the order to arrangement of the treatments with respect to the graph of the PCA; it means that in Figure 11, to this specific compound, the treatments are ordered from the highest to the lowest concentration from left to right; however, the compounds 3-phenyl-2-propenal, butyl-2-butiryloxypropanoate, and 5-pentyloxolan-2-one, which are in the positive part of axis 1 in Figure 12, have inverse behavior; that is, higher concentration treatments are right and its concentration is decreasing as they are located more to the left of the graph.

With this, we can say that the compounds causing the synergism are 3-phenyl-2-propenal, butyl-2-butiryloxypropanoate, and 5-pentyloxolan-2-one. The DFA reinforced the statement in the PCA; that is, treatments are distinct and there is no error of confusion between treatments.

## 4. Conclusion

The fermentation of blackberry juice with* Saccharomyces cerevisiae *has potential to be produced at industrial scale. Thirty five aromatic compounds were identified by their higher concentration in blackberry spirit produced in monoculture, mixed culture, and monoculture blackberry wine mixture. The differences in aromatic profile principally rely on the proportions in aromatic compounds and not on the number of these compounds. The multivariate analysis, principal component analysis (PCA), and factorial discriminant analysis (DFA) permit to demonstrate the synergism between the strains during fermentation process.

## Figures and Tables

**Table 1:** Strains combinations for mixed culture.

*Saccharomyces cerevisiae *strains			

M1	ICV K1 (25 mg/L)	+	T306 (25 mg/L)
M2	ICV K1 (25 mg/L)	+	CM4457 (25 mg/L)
M3	ICV K1 (25 mg/L)	+	L3574 (25 mg/L)
M4	T306 (25 mg/L)	+	CM4457 (25 mg/L)
M5	T306 (25 mg/L)	+	L3574 (25 mg/L)
M6	CM4457 (25 mg/L)	+	L3574 (25 mg/L)

**Table 2:** Aromatic compounds identified in the blackberry spirit.

Alcohols	Aldehydes
Ethanol 3-Methyl-1-butanol 3,7 Dimethyl-6-octen-1-ol 2-Methyl-5-(1-methylethyl)-phenol 2,6 Bis (1,1 dimethylethyl)-4-methylphenol	Benzaldehyde 3-Phenyl-2-propenal Ethyl-3-methyl-3-phenyl-oxirane-2-carboxylate 5-Pentyloxolan-2-one

Terpenes	Fatty acids

3,7-Dimethyl-2,6-octadien-1-ol 3,7-Dimethyl-1,6-octadien-3-ol 5-Methyl-2-isopropyl cyclohexane-1-ol	Decanoic acid Dodecanoic acid

Esters	

3-Methylbutyl acetate 2-Methylpropyl butanoate 3-Methylbutyl propanoate Butyl butanoate 1-Methylbutyl propanoate Phenylmethyl formiate 3-Methylbutyl butanoate Allyl hexanoate 3-Methylbutyl-3-methylbutanoate Ethyl octanoate	2-Phenylethyl acetate Phenylmethyl propanoate 3-Methylbutyl-hexanoate Hexyl acetate Butyl-2-butiryloxypropanoate Ethyl dodecanoate Phenylmethyl butanoate Phenylmethyl benzoate Ethyl hexadecanoate

Ketones	

(E)-4-(2,6,6 Trimethyl-2-cyclohexene-1-il)-3-buten-2-one	
(E)-4-[(5R)-5,6,6-Trimethylcyclohexene-1-il]but-3-en-2-one	

**Table 3:** Average concentration for the principal aromatic compounds in the blackberry spirits.

Groups of blackberry spirit	Concentration (*µ*g/L)
Monoculture	334.336 ± 84.06
Spirits mixture	322.098 ± 67.40
Mixed cultures	366.055 ± 72.73

**Table 4:** Confusion matrix resulting from the different spirits DFA. Monoculture (MC), spirits mixtures (SM), and mixed cultures (MC).

	MC	SM	MC
MC	10	0	2
SM	3	14	1
MC	1	1	16

**Table 5:** Principal aromatic compounds concentration for the different blackberry spirits.

Blackberry spirit	Concentration (*µ*g/L)	
	1-Methylbutyl propanoate	Decanoic acid
Monoculture CM4457	3.91	1.43
Monoculture L3574	4.64	0.96
Spirit mixture	4.76	0.99
Mixed culture	7.56	1.83

**Table 6:** Principal aromatic compounds average concentration for the different blackberry spirits made with the strains ICV K1 y T306.

Blackberry spirit	Concentration (*µ*g/L)
ICV K1	4.53
T306	9.22
Spirits mixture	6.41
Mixed culture	7.90

**Table 7:** Principal aromatic compounds concentration for the blackberry spirits made with the strains ICV K1 y CM4457.

Blackberry spirits	Concentration of aromatic compounds (*µ*g/L)						
	3-Methyl-1-butanol	3-Methylbutyl propanoate	3,7-Dimethyl-2,6-octadien-1-ol	3-Methylbutyl butanoate	3-Phenyl-2-propenal	Dodecanoic acid	3-Methylbutyl-3-methylbutanoate
CM4457	424.34	0.353	16.104	0.000	7.357	4.293	0.166
ICV K1	349.72	0.496	5.618	0.183	4.099	0.187	0.040
Spirits mixture	371.77	0.507	9.400	0.066	5.940	1.225	0.075
Mixed culture	599.35	0.246	16.110	0.019	13.220	6.094	0.033

**Table 8:** Confusion matrix resulting from the strains CM4457 and ICV K1 DFA. Monoculture (MC), spirits mixtures (SM), and mixed cultures (MC).

	CM4457	ICVK1	SM	MC
CM4457	3	0	0	0
ICV K1	0	3	0	0
SM	0	0	3	0
MC	0	0	0	3
